# A Game Theoretic Optimization Method for Energy Efficient Global Connectivity in Hybrid Wireless Sensor Networks

**DOI:** 10.3390/s16091380

**Published:** 2016-08-29

**Authors:** JongHyup Lee, Dohyun Pak

**Affiliations:** Department of Mathematical Finance, Gachon University, 1342 Seongnamdaero, Seongnam-si 13120, Korea; jonghyup@gachon.ac.kr

**Keywords:** game theory, resource allocation, CDMA, TDMA

## Abstract

For practical deployment of wireless sensor networks (WSN), WSNs construct clusters, where a sensor node communicates with other nodes in its cluster, and a cluster head support connectivity between the sensor nodes and a sink node. In hybrid WSNs, cluster heads have cellular network interfaces for global connectivity. However, when WSNs are active and the load of cellular networks is high, the optimal assignment of cluster heads to base stations becomes critical. Therefore, in this paper, we propose a game theoretic model to find the optimal assignment of base stations for hybrid WSNs. Since the communication and energy cost is different according to cellular systems, we devise two game models for TDMA/FDMA and CDMA systems employing power prices to adapt to the varying efficiency of recent wireless technologies. The proposed model is defined on the assumptions of the ideal sensing field, but our evaluation shows that the proposed model is more adaptive and energy efficient than local selections.

## 1. Introduction

Recent advances in the Internet of Things (IoT) accelerate the deployment of wireless sensor nodes, since sensor nodes will play roles as important observers and collectors in the IoT environments. As we actively install wireless sensor networks (WSNs) in the real field, we are realizing that the previous research assumes overly ideal WSNs, where identical sensor nodes compose a large-scale wireless mesh network. However, a large wireless mesh network suffers the severe degradation of bandwidth and low connectivity for the connections of multiple wireless hops [1]. The more realistic approach for deploying WSNs is to partition a whole WSN into smaller networks. A partitioned WSN forms a cluster that consists of a handful of sensor nodes. Sensor nodes gather information and report it to a sink node through a head node of the cluster, the cluster head. The cluster head is liable for maintaining global connectivity to the sink node or other cluster heads. In this sense, it may have two network interfaces: one for the global connectivity (inter-cluster communication) and the other for cluster members (intra-cluster communication). When only cluster heads have two different network interfaces, we call the WSN a “hybrid WSN”.

For intra-cluster communication, sensor nodes can use low-power, but coverage-limited wireless communication methods, e.g., ZigBee, 6LowPAN and Wireless HART. For inter-cluster communication, cellular networks of high coverage are a practical option. The latest improvements in cellular networks for IoT devices, such as LTE-M, Lora and Sigfox, encourage small devices to employ cellular networks.

The hybrid WSN is a realistic solution for a number of WSN applications, but it makes cluster heads’ lifetime a critical factor. The cluster heads consume more energy than other members for having a higher communication load to forward traffic through the cellular network. Moreover, a cluster head basically takes a greedy approach to maintain stable connections for its cluster members. Therefore, it is important to coordinate cluster heads to select appropriate base stations that can satisfy as many node as possible.

In this paper, we propose a new method for maintaining global connectivity by allocating cluster heads to optimal base stations with respect to energy efficiency and availability in hybrid WSNs. For the purpose, game theory [2] presents a reasonable way for modeling interactions between self-interested users and coordinating their strategies. It also plays a role to optimally allocate limited resources reacting to behaviors of the other parties [3,4]. Thus, we apply game theory to allocate limited cellular base stations to cluster headers. Our game theoretic model provides an optimal base station allocation, and the cluster head can be coordinated to efficiently achieve global connectivity.

The contributions of this paper can be summarized as follows:

1. For efficient resource management in hybrid WSNs, we present an optimization method based on game theory. By using the proposed model, cluster heads connecting cellular networks can find the optimal base station under the given cost function.
2. We formulate different characteristics of cellular networks into a cost function for game theoretic models. To apply the divergent advances in cellular networks, e.g., femtocells, LTE-M, Sigfox, etc., we set a parameter that represents power price per a communication unit, such as a slot or a packet. Depending on wireless multiplexing technologies, base stations have constraints in available slots or SNIR (signal to noise plus interference ratio). Thus, we provide a cost function for TDMA/FDMA and CDMA, respectively.
3. We perform simulation and analysis on the proposed model with respect to efficiency, flexibility and resilience against changes (mobility). From the result, we can find interesting facts, such as the optimal coordination is important for lowering power price, i.e., using low power wireless technologies.

## 2. System Model and Motivations

### 2.1. Two-Tier Hybrid WSN

In cluster-based WSNs, the cluster members are located closely. Intra-cluster connectivity can be easily achieved even with energy-efficient wireless networks. On the other hand, the proximity of clusters is undetermined. A battlefield is a good example of a cluster-based WSN. A soldier installs a cluster of bio-medical sensors on his/her body. All of the member sensors of a single cluster are located within a few meters. However, each soldier independently moves in the field, so its cluster does. Thus, the cluster heads need another communication method of wider-range coverage for inter-cluster connectivity.

**Figure 1:**
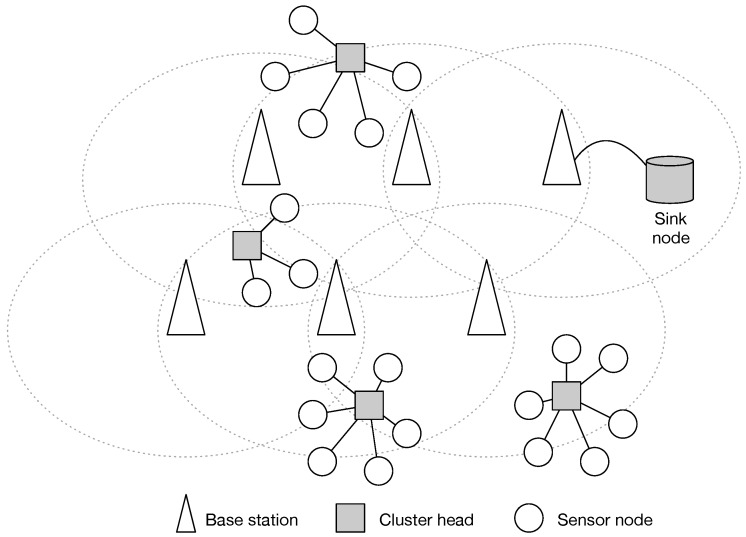
Cluster head in hybrid wireless sensor networks.

A network interface of hybrid WSNs is represented as a tier, since it provides independent communication. In this paper, we focus on the upper tier, which is for inter-cluster communication. It is reasonable for the cluster heads to employ cellular communications. Once multiple base stations cover a whole field, the global connectivity of clusters is achievable. Figure 1 shows the structure of two-tiered WSNs.

Note that, in hybrid sensor networks, we differentiate base station from sink node. The base stations provide connectivity to cellular users. the sink node is the control tower of a sensor network, as well as the end point of sensing data.

### 2.2. Background

Game theory: Game theory is a tool in modeling interactions between self-interested users (game theory is summarized, e.g., in [2]). Under a game equilibrium, all agents operate optimally, and therefore, all resources are allocated efficiently. The game equilibrium is not necessarily the same as the global optimum. Johari and Tsitsiklis [5] analyze the loss in a network resource allocation game and show that the selfish behaviors of the network users lead to an aggregate utility, which is no worse than 75% of the maximum possible aggregate utility. Specifically, in our model, we consider the optimal base-station assignment in the cellular system with fixed numbers of base-stations and users, where each user minimizes his/her own cost.

Cellular system: The cellular system is categorized according to the multiple access scheme or frequency gain process [6,7,8]. Frequency division multiple access (FDMA) and time division multiple access (TDMA) are used in GSM-based systems, e.g., UMTS, in a mixed form. In FDMA, frequencies are assigned to users, and therefore, the larger the number of users in the FDMA system, the larger the number of available frequencies must be. On the other hand, TDMA allows several users to share the same channel. Each user shares the common channel and is assigned to its own burst within a group of bursts, called a frame. The availability of the radio spectrum and time frame limits the number of users in the FDMA/TDMA system. In code division multiple access (CDMA), each user is assigned a unique code sequence that it uses to encode its information-bearing signal. The receiver, knowing the code sequence of the users, decodes a received signal after reception and recovers the original data. TDMA and FDMA are interchangeable. Both split the total channel into disjoint slots, such as time frames or frequency bands. However, CDMA users employ overlapped channels, but disjoint code sequences. Thus, CDMA has the near-far problem, where communication performance depends on the signal strength of base stations and other users.

### 2.3. Cellular Model for Cluster Heads

We consider a set {1,⋯,a} of cluster heads and a set {1,⋯,b} of base stations. Each admitted cluster head to a base station consumes resources of the base station and pays a price in return. In this section, we discuss these models and provide some intuitive pricing properties for the models.

In a cellular system, the covering area of an operator is divided into cells. A cell corresponds to the covering area of one transmitter or a small collection of transmitters. Here, we consider a single-service transmitter and call it a base station. The size of a cell is determined by the base station’s power.

In a single-service TDMA (or FDMA) system, the time frame (frequency band) assigned to a cell *k* is of a fixed length $S_{k}$ (capacity), and upon admission, each cluster head is assigned a frequency band (time slot) of width *u*. Let $N_{k}$ denote the number of calls connected to the base station *k*. Then, $N_{k}u$ is the total frequency band needed for base station *k*, and it has to be less than the allocated capacity $S_{k}$. Therefore, we suggest that the price of admission by base station *k* is low if $N_{k}u\leq S_{k}$, since there is free capacity, and the price is very high or even infinite if $N_{k}u>S_{k}$. In other words, the connection price at each base station depends only on the number of cluster heads connected to the base station.

In a single-service CDMA system, on the other hand, each admitted cluster head is assigned one node from the base station. If cluster head *i* transmits with power *P*, the pre-decoding received power at the base station is $P=h_{ik}P$, where $h_{ik}$ is the channel gain between cluster head *i* and base station *k*. Admissions are performed as long as it is possible for the system to achieve a target value *γ* for the received SNIR for the admitted cluster heads (see, e.g., [9]). In other words, the price of connection to a base station depends on the achieved SNIR, and it is low if the received SNIR is greater than the target value *γ*, while the price is very high or infinite if the SNIR is less than *γ*. In CDMA systems, unlike TDMA systems, the received SNIR at base station *k* depends on the transmission power of all cluster heads that happen to be close to the base station *k*, no matter to which base station they are connected. That is, the connection price of one cluster head in CDMA system is affected by other cluster heads’ activities. This fact has the following implications. First, this implies that the price of a base station cannot be solely determined by the load connected to it; second, it implies that the change of assignment for a cluster head that is extremely close to the base station *k* to a far base station not only causes an increase in the price of base station *k*, but it also drives up the price of the far station. Note that the channel gain process of the CDMA system is different from TDMA or FDMA systems, and SNIR is only used in the CDMA system.

### 2.4. Optimization Problems for Cluster Heads

The optimization goal for cluster heads is two-fold: (1) all cluster heads should be able to find an optimal base station that minimizes the energy consumption; and (2) all base stations should be available for serving cluster heads. In other words, cluster heads need to maintain both reliable and energy-efficient connection to a base station. However, due to self-interested cluster heads and the near-far problem, the optimal solution is dependent on other cluster heads’ strategies. Note that we assume that each cluster head cannot learn the status of other cluster heads. Scanning a whole frequency band to find out the population of other cluster heads is impractical due to energy consumption and the spreading factors of CDMA. Message exchanges between cluster heads and the sink node are also protected by end-to-end encryption. Thus, we assume that the information structure of game theory is not broken, which can be caused by leaking private information [10], so that the instability in the results is avoidable. In the following section, we present our model based on game theory to assign optimal base stations for self-interested cluster heads.

## 3. Optimal Base Station Selection of Hybrid Cluster Heads

### 3.1. Optimization Process Overview

The proposed method computes the feasibility of base stations for each cluster head based on information collected from cluster heads. We denote the feasibility of a cluster head *i* by a vector $\pi_{i}=\left(\pi_{i,1}\cdots \pi_{i,b}\right)$ of probability distribution. A cluster head then chooses a base station to connect to depending on its feasibility vector. Since the feasibility vector does not give a deterministic solution, cluster heads may have their own strategies to select appropriate base stations (Section 3.4). The feasibility vector is updated at every time interval, T. For accurate results, we assume that a sink node has pre-knowledge of base stations’ location and multiplexing type, such as TDMA, FDMA and CDMA. The overall process is as follows:

1. Cluster heads identify reachable (within communication range) base stations, $V_{i}$ (of cluster head *i*), and report the information to a sink node with their location $L_{i}$.
2. The sink node gathers information from cluster heads and applies the proposed game theory model with the pre-knowledge on the base stations.
3. For each cluster head, the sink node calculates a feasibility vector and reports it.
4. A cluster head selects a base station based on its strategy with the feasibility vector.
5. Repeat the whole process at every time interval T.

We explain the detailed process for the cluster heads and the sink node in Section 3.4.

### 3.2. Game Theoretic Models for TDMA and FDMA Systems

In this section, we formulate game models for TDMA and FDMA systems. For simplicity, we use only the name TDMA to represent both TDMA and FDMA, since they are dual between the time slot and the frequency band. As shown in Section 3.1, each cluster head individually sends its periodic report to the sink node and the sink node computes the feasibility through the model in Section 3.2 and Section 3.3. Since wireless communications in cellular systems is synchronously operated, the cluster heads simultaneously find the base station. Thus, we assume that cluster heads’ behaviors can be modeled as parallel processes. First, we introduce cluster heads’ cost functions that have two terms: unit capacity price, *c*, and power cost, *C*. Second, we derive the game model based on the cost function. To do so, we make assumptions to model the target sensing field. The assumptions simplify the details of the real world, but reflect general cellular systems.

The total number of connections for base station *k*, $N_{k}$, can be represented by the number of cluster heads that connected to the base station *k*. Therefore, the total capacity demands of base station *k* is given by $u\Sigma_{i\in \{1,\cdots ,a\}}I_{i,k}\left(t\right)$, where *u* is the capacity units used by one cluster head and indicator:

(1) $$I_{i,k}\left(t\right)=\left\{\begin{array}{cc} 1, & \text{if}\;\text{a}\;\text{cluster}\;\text{head}\;i\;\text{is}\;\text{using}\;\text{the}\;\text{base}\;\text{station}\;k\;\text{at}\;\text{time}\;t\\ 0, & \text{Otherwise} \end{array}\right.$$

We assume that the capacity of station *k*, $S_{k}$, is constant. The capacity price of base station *k* is low if $u\Sigma_{i\in \{1,\cdots ,a\}}I_{i,k}\left(t\right)<S_{k}$, and the price is infinite if $u\Sigma_{i\in \{1,\cdots ,a\}}I_{i,k}\left(t\right)\geq S_{k}$, since the delay of over-capacity base stations increase indefinitely [11]. Therefore, we assume that $u\Sigma_{i\in \{1,\cdots ,a\}}I_{i,k}\left(t\right)\in \left[0,S_{k}\right)$ and that the unit capacity price is given by Assumption 1.

**Assumption 1.**  *The unit capacity price of base station k is given by:*

(2) $$c_{k}\left(t\right)=\left\{\begin{array}{cc} \frac{1}{S_{k}-u\Sigma_{i\in \{1,\cdots ,a\}}I_{i,k}\left(t\right)}, & if\;S_{k}>u\Sigma_{i\in \{1,\cdots ,a\}}I_{i,k}\left(t\right)\\ \infty , & otherwise \end{array}\right.$$

*for all t∈[0,T] and k∈{1,⋯,b}.*

Now, we assume that each cluster head is spending power according to the following model.

**Assumption 2.**  Power usage of cluster head i∈{1,⋯,a} corresponding to base station k∈{1,⋯,b} depends on the distance between the node (cluster head) and the base station. Thus, the power usage C(·) is an increasing function of the distance $m_{i,k}\left(t\right)$.

For example, the cost function for a power controlled system with target power $P_{0}$ is given by $C\left(m\right)=P_{0}m^{4}$. Note that we cannot specify all of the specific terrain and geographical characteristics of target fields in the model. Thus, we assume that the distance of Assumption 2 is a normalized metric, presuming an ideal, flat field without any external interference, except entities of the model.

**Assumption 3.**  The power price is constant.

Combining Assumptions 2 and 3, we get that cluster head *i*’s power cost at base station *k* is given by $pC(m_{i,k}\left(t\right))$, where *p* is the power price and is given depending on the wireless communication technologies.

The objective of cluster head *i* is to minimize its costs by selecting the optimal base station. That is,

(3) $$J_{i}\left(t\right)=\min_{k\in \{1,\cdots ,b\}}pC\left(m_{i,k}\left(t\right)\right)+c_{k}\left(t\right)$$

For the game formulation, we consider the feasibility of cluster heads for base station selections. That is, the objective function for cluster head *i* is this game system as follows,

$$J_{i}\left(t\right)=\min_{\pi_{i}}\Sigma_{k\in \{1,\cdots ,b\}}\left\{\pi_{i,k}\left(t\right)pC\left(m_{i,k}\left(t\right)\right)+\frac{1}{S_{k}-u(\pi_{i,k}\left(t\right)+\Sigma_{z\in \{1,\cdots ,a\}\backslash i}\pi_{z,k}\left(t\right))}\right\}$$

where $\pi_{i}$ is the feasibility vector of cluster head *i*; $\Sigma_{k\in \{1,\cdots ,b\}}\pi_{i,j}\left(t\right)=1$ and $\pi_{i}\geq 0$ for all i∈{1,⋯,a}.

There are two factors in each cluster head’s objective function Equation (3): power cost and capacity cost. According to Assumption 2, power cost depends on the distance between the cluster head and the base station. However, the capacity cost is infinite when the number of connected users exceeds the capacity. Therefore, to minimize overall cost, the capacity cost part has to be finite, and due to this property, the TDMA system is often referred to as having no near-far problem.

Let $\lambda_{0},\lambda_{1},\cdots ,\lambda_{b}$ be the Kuhn–Tucker multipliers for the constraints so that the Lagrangian takes the form:

(4) Li(t)=minπiΣk∈{1,⋯,b}πi,k(t)pC(mi,k(t))+1Sk−u(πi,k(t)+Σz∈{1,⋯,a}\iπz,k(t))1Sk−uπi,k(t)+Σz∈{1,⋯,a}\iπz,k(t)+λ0(1−Σk∈{1,⋯,b}πi,k(t))+Σk∈{1,⋯,b}λkπi,k(t).

Differentiating with respect to $\pi_{i,j}\left(t\right)$ gives:

$$\pi_{i,k}\left(t\right)=\frac{1}{u}S_{k}-\Sigma_{z\in \{1,\cdots ,a\}}\pi_{z,k}\left(t\right)-\frac{1}{u}\sqrt{\frac{u}{-pC\left(m_{i,k}\left(t\right)\right)+\lambda_{0}-\lambda_{k}}}$$

or:

(5) $$u\pi_{i,k}\left(t\right)=S_{k}-u\Sigma_{z\in \{1,\cdots ,a\}}\pi_{z,k}\left(t\right)-\sqrt{\frac{u}{-pC\left(m_{i,k}\left(t\right)\right)+\lambda_{0}-\lambda_{k}}}$$

This implies: “expected *k*’-th base station’s capacity usage” = “expected free capacity in base station *k*” -“penalty from the condition $\Sigma_{k\in \{1,\cdots ,b\}}\pi_{i,k}\left(t\right)=1$ and $\pi_{i}\geq 0$”.

We denote the solution to Equation (5) as $\pi_{i}^{*}$, and then, the system’s equilibrium is $\pi^{*}=\left(\pi_{1}^{*}\cdots \pi_{n}^{*}\right)$ where each element is corresponding to Equation (5). Note that it is for a Nash equilibrium, where no player can increase his/her expected payoff by using a different strategy (the base station assignment is a parallel process for cluster heads and occurring globally; thus, it is not subgame perfect Nash equilibrium). The equilibrium can be solved iteratively as follows by using the Tâtonnement process (see [2]). We assume that the iterations of the Tâtonnement process end when the sum of changes goes below a saturation threshold, S.

1. Set the initial value for $\pi_{i}$.
2. Store $\pi^{*}$ as $\pi_{o}^{*}$.
3. For all i∈{1,⋯,a}, solve Equation (5) by assuming that the cluster heads in the set {1,⋯,a}\{i} keep their solutions fixed.
4. Repeat Steps 2 and 3 until $|\pi_{o}^{*}-\pi^{*}|<S$.

As mentioned in Section 1, this game has a nice stability property [12,13], and we show it in Section 4. Due to the characteristics of the Tâtonnement process, we can get a single equilibrium from this approach.

Under the game equilibrium, the cluster head *i*’s cost is given by:

(6) $$J_{i}\left(t\right)=\Sigma_{k\in \{1,\cdots ,b\}}\left\{\pi_{i,k}^{*}\left(t\right)pC\left(m_{i,k}\left(t\right)\right)+\frac{1}{S_{k}-u\left(\pi_{i,k}^{*}\left(t\right)+\Sigma_{z\in \{1,\cdots ,a\}\backslash i}\pi_{z,k}^{*}\left(t\right)\right)}\right\}$$

where $\pi_{i}^{*}=\left(\pi_{i,1}^{*}\cdots \pi_{i,b}^{*}\right)$ describes cluster head *i*’s optimal probability distribution of selecting different base stations.

### 3.3. Game Theory Formulation for CDMA System

A CDMA system is based on sharing the bandwidth among different users at any instance of time, rather than breaking the total bandwidth or time units into smaller dedicated bandwidths or time-slots assigned to each user. The advantage of such a system is in the simplicity of implementing a scheduling algorithm. As in the last section, we first introduce the cost functions (each cost function is again the sum of *c* and p·C), and then, after that, formulate the game model based on the cost functions.

We make the following assumption, similar to the previous section, with regard to the power usage of a cluster head.

**Assumption 4.**  Power usage of cluster head i∈{1,⋯,a} assigned to base station k∈{1,⋯,b}, $C_{i,l}$ is controlled in order to compensate the channel attenuation, i.e., $C_{i,k}=P_{0}/g_{i,k}$ where $P_{0}$ is the target power and $g_{i,k}$ is the channel gain. Furthermore, we assume that the channel gain, $g_{i,k}$, depends on the distance between the cluster head and the base station, i.e., $g_{i,k}$ is high when the cluster head i is close to base station k. Thus, the power usage $C_{i,k}$ is an increasing function of the distance $m_{i,k}\left(t\right)$.

For example, the cost function for a power controlled system with target power $P_{0}$ and where the channel gain follows the fourth power law (see, e.g., [9]) is given by $C\left(m\right)=P_{0}m^{4}$.

Combining Assumptions 3 and 4, we get that agent *i*’s power cost at base station *k* is given by $pC(m_{i,k}\left(t\right))$ where *p* is the power price.

Furthermore, in CDMA systems, cluster head *i*’s usage of each base station *k* is determined by the received signal to noise ratio, $SNIR_{i,k}$. As introduced in Section 2.3, it is desirable for the cluster head *i*’s connection price to base station *k* to be low if $SNIR_{i,k}>\gamma$ and to be infinite if $SNIR_{i,k}\leq \gamma$. Note that SNIR is only used in the CDMA system.

Therefore, we assume that $SNIR_{i,k}\in \left(\gamma ,\infty \right)$ and that the unit capacity price is given by the following assumption.

**Assumption 5.**  *For cluster head i, the connection cost to base station k is given by:*

(7) $$c_{k}\left(t\right)=\left\{\begin{array}{cc} \frac{1}{SNIR_{i,k}-\gamma }, & if\;SNIR_{i,k}>\gamma \\ \infty , & otherwise \end{array}\right.$$

The main difference between a CDMA system and a TDMA system is the presence of externality in the CDMA system. In other words, in order for a new cluster head to achieve the desirable SNIR, the system has to consider both the load of the used base station and the load of the neighboring cells whose load plays an externality role for the used base station. In a multi-cell CDMA system, the received SNIR for cluster head *i* at base station *k* equals (see [9], Chapter 8):

(8) $$SNIR_{i,k}=\frac{N_{c}P_{0}G_{0}}{\eta +P_{in}^{r}\left(k\right)+P_{ex}^{r}\left(k\right)}$$

where $N_{c}$ is the number of sectors in each cell, $P_{0}$ is the received power of each cluster head at the used base station (due to the power control mechanism), $G_{0}$ is the spreading gain, *η* is the background additive noise in the spread bandwidth and $P_{in}^{r}\left(k\right)$ and $P_{ex}^{r}\left(k\right)$ are the internal (cluster heads connected to base station *k*) and external (all other cluster heads) interference terms received at the base station *k*, respectively. A cell is typically sectorized into three sectors to achieve rapid frequency reuse (see [14] for optimum sectorization for the CDMA system). We assume that at the parameters $N_{c}$, $P_{0}$ and *η* are constants. Using Assumption 4, we have:

(9) $$\begin{array}{cc} SNIR_{i,k} & =\frac{N_{c}P_{0}G_{0}}{\eta +P_{0}\left(\right|J_{k}\left|-1\right)+\Sigma_{j\neq i}\Sigma_{j\in J_{l}}C_{j,l}g_{j,k}}=\frac{N_{c}P_{0}G_{0}}{\eta +\Sigma_{j\neq i}\Sigma_{l=1}^{b}g_{j,k}C_{j,l}I_{j,l}}\\ & =\frac{N_{c}P_{0}G_{0}}{\eta +P_{0}\Sigma_{j\neq i}\Sigma_{l=1}^{b}\left(C_{j,l}/C_{j,k}\right)I_{j,l}} \end{array}$$

where $J_{l}$ is the set of cluster heads connected to base station *l* and $C_{j,l}$, $g_{j,l}$ and $I_{j,l}$ are defined in Assumption 4 and Equation (1). We assume that there is a perfect sectorization of each cell. Based on Equation (9), Assumption 5 can be re-written as follows.

**Assumption 5-1.**  *For cluster head i, the connection cost to base station k is given by:*

(10) $$c_{k}\left(t\right)=\left\{\begin{array}{cc} \frac{1}{S_{k}-u\Sigma_{j\neq i}I_{j,k}}, & if\;S_{k}>u\Sigma_{j\neq i}I_{j,k}\\ \infty , & otherwise \end{array}\right.$$

*where $u=\gamma P_{0}$ and $S_{k}=N_{c}P_{0}G_{0}-\gamma \eta -u\Sigma_{j\neq i}\Sigma_{l\neq k}\left(g_{j,k}/g_{j,l}\right)I_{j,l}$.*

The objective of cluster head *i* is to minimize his/her costs by selecting the optimal base station. That is,

(11) $$J_{i}\left(t\right)=\min_{k\in \{1,\cdots ,b\}}pC\left(m_{i,k}\left(t\right)\right)+c_{i,k}\left(t\right)$$

As in the TDMA system, the objective function Equation (11) has two factors: power cost and connection cost. According to Assumptions 2, 4 and 5, power usage, as well as the connection cost depends on the distance between the cluster head and the base station. Hence, the optimization problem in the CDMA system is different from the TDMA system, even though the objective function has a similar structure.

For the game formulation, we consider each cluster head’s optimal probabilities for base station selections. That is, the objective function for cluster head *i* in this game system is as follows,

(12) $$J_{i}\left(t\right)=\min_{\pi_{i}}\Sigma_{k\in \{1,\cdots ,b\}}\left\{\pi_{i,k}\left(t\right)pC\left(m_{i,k}\left(t\right)\right)+\frac{1}{S_{k}^{\pi }-u\Sigma_{j\neq i}\pi_{j,k}\left(t\right)}\right\},$$

where $\pi_{i}=\left(\pi_{i,1}\cdots \pi_{i,k}\right)$, $S_{k}^{\pi }=N_{c}P_{0}G_{0}-\gamma \eta -u\Sigma_{j\neq i}\Sigma_{l\neq k}\left(g_{j,k}/g_{j,l}\right)\pi_{j,l}$, $u=\gamma P_{0}$ and $\Sigma_{k\in \{1,\cdots ,b\}}\pi_{i,k}\left(t\right)=1$ and $\pi_{i}\geq 0$ for all i∈{1,⋯,a}.

By using the same technique as in Section 3.2, the agent *i*’s cost under the game equilibrium can be written as:

(13) $$J_{i}\left(t\right)=\min_{\pi_{i}}\Sigma_{k\in \{1,\cdots ,b\}}\left\{\pi_{i,k}^{*}\left(t\right)pC\left(m_{i,k}\left(t\right)\right)+\frac{1}{S_{k}^{\pi^{*}}-u\Sigma_{j\neq i}\pi_{j,k}^{*}\left(t\right)}\right\},$$

where $\pi_{i}^{*}=\left(\pi_{i,1}^{*}\cdots \pi_{i,k}^{*}\right)$, a set of feasibility vectors, which describes agent *i*’s optimal probabilities of selecting different base stations.

### 3.4. Base Station Selection Strategies

The sink node computes the feasibility vector for each cluster head and delivers it to cluster heads. Based on the feasibility vector, a cluster head chooses a base station to which to connect. The game theory model does not give a deterministic solution, but a possibility. Moreover, since we assume crowded wireless networks, the selected base station may be unavailable due to high load caused by other nodes. Unless only a single base station is definitely feasible (i.e., $\pi_{i,k}=1$), a cluster head has a selection strategy. First, select a dominantly-feasible base station. If the feasibility of a base station is greater than a handoff threshold F, then we call it as the “primary base station,” and the cluster head first tries to connect to the base station since it is obviously more advantageous than others. If there is no primary base station, a cluster head randomly chooses a base station as the probability given by the feasibility vector. Combining the overview in Section 3.1 and the strategy, we can summarize the detailed process for the cluster heads and the sink node in Algorithms 1 and 2, respectively.

**Algorithm 1:** Process of the *i*-th cluster head.
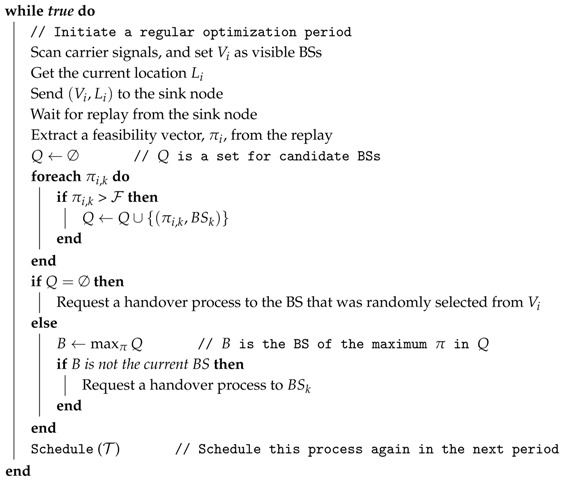


**Algorithm 2:** Process of the sink node.
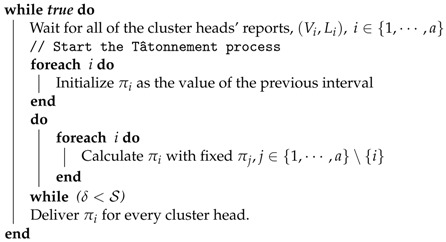


## 4. Evaluations

In this section, we evaluate the proposed game model by simulations. Since TDMA and FDMA are interchangeable, we performed two types of simulations with TDMA and CDMA base stations. For each simulation, we compare the energy cost of all cluster heads depending on the optimizing coordination of the proposed method.

### 4.1. Simulation Setup

**Figure 2:**
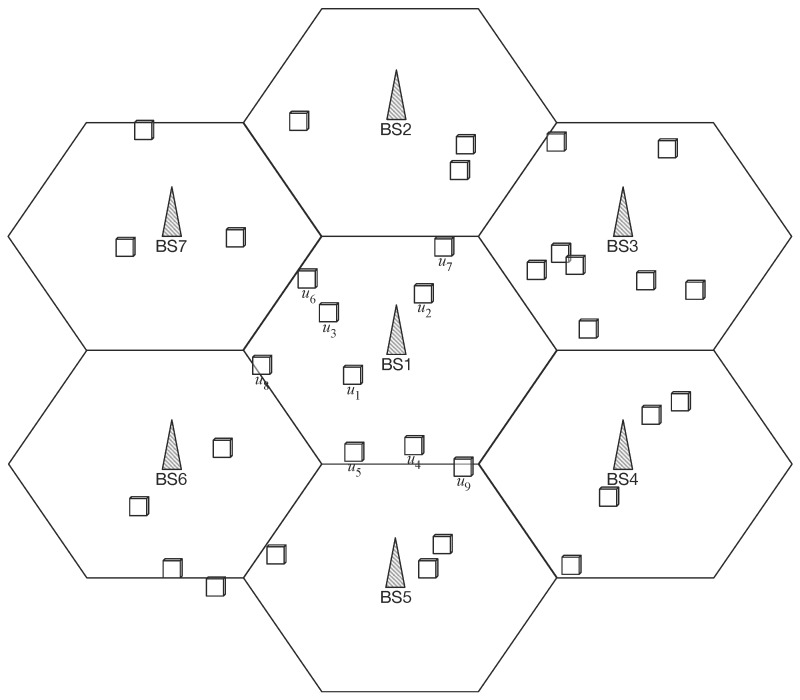
Simulation setup.

We instantiate a hybrid sensor network with seven base stations and 35 randomly-located clusters. Figure 2 shows the simulation setup. Since the optimal base station assignment is critical in crowded, high-load networks, we allocate nine cluster heads in the center cell for Base Station 1 (BS1) and focus on the result of the nine cluster heads.

The target field for simulation is a flat area of 1200 m × 1200 m. The distance between base stations is 400 m. We ran the simulation 20 times to get the average of the results (when we need the changes over iterations, we use a single instance from the simulation results). For every interval T, we relocated cluster heads at the random position of different distance from the base station. For better readability, we always assign each cluster head’s number according to the distance from the base station, e.g, the nearest cluster head has number 1 and the farthest cluster head has number 9 for BS1. Inter-cell movement is simulated with respect to the resilience of the proposed model. In this simulation setup, we consider only cluster heads for simplicity, but this can be applied to practical scenarios, e.g., a battlefield of soldiers wearing bio-medical sensors in Section 2.

### 4.2. TDMA Base Station

In the TDMA system, the cluster head *i* has objective function Equation (3), and the cost under the equilibrium is given by Equation (6). We assume the target power $P_{0}=\frac{10^{0.8}}{50}$. The cost function for the TDMA system is given by $C\left(m_{i,k}\right)=P_{0}m_{i,j}^{4}$, where $m_{i,k}$ is the distance between cluster head *i* and the base station *k*. From the assumed parameter values, each base station’s capacity is 5.5 cluster heads using one unit channel including guards ($S_{k}=5.5$, u=1), and the cost is infinite when a base station has more than its capacity. We set the handoff threshold F as 0.7 and the initial value for $\pi_{i,k}$ as follows:

(14) $$\pi_{i,k}=\left\{\begin{array}{cc} 1, & \text{if}\;k\;\text{is}\;\text{the}\;\text{nearest}\;\text{base}\;\text{station}\;\text{for}\;\text{cluster}\;\text{head}\;i\\ 0, & \text{otherwise} \end{array}\right.$$

for all i∈{1,⋯,35} and k∈{1,⋯7}.

To evaluate our game model, we set an intuitive coordination scheme, denoted by NearestFirst. We discuss other recent schemes in Section 5. The schemes in Section 5, however, are difficult to directly compare with the proposed model because the cost functions are incompatible. Therefore, we define NearestFirst, and it resembles the behavior of user devices in cellular systems. In the NearestFirst scheme, a cluster head first connects to the nearest base station until the base station becomes unavailable due to the high load. Once the base station is unavailable, the cluster head randomly selects another base station among its reachable base stations.

**Figure 3:**
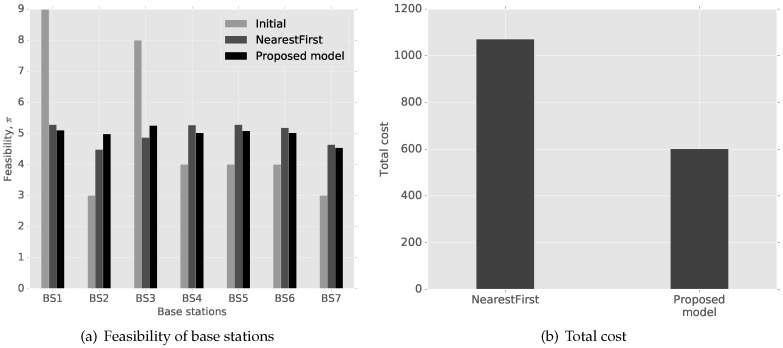
Feasibility of base stations and the total cost of cluster heads with TDMA systems.

Figure 3 shows the changes in the feasibility of base stations and the total cost of cluster heads. Note that $\Sigma_{i}\pi_{i,k}$ is the total sum of all cluster heads’ feasibility to use base station *k*. The total sum of all cluster heads’ cost is shown in Figure 3b. In our evaluation, we assume a crowded network, i.e., high loads on base stations. Since Base Stations 1 and 3 have more cluster heads than the capacity limit, the capacity cost from the two base stations is infinite by Assumption 1. After the initial assignment, NearestFirst and the proposed model distribute cluster heads’ connections to other base stations, as shown in Figure 3a. However, the total cost of our game model is significantly lower by 43%.

**Figure 4:**
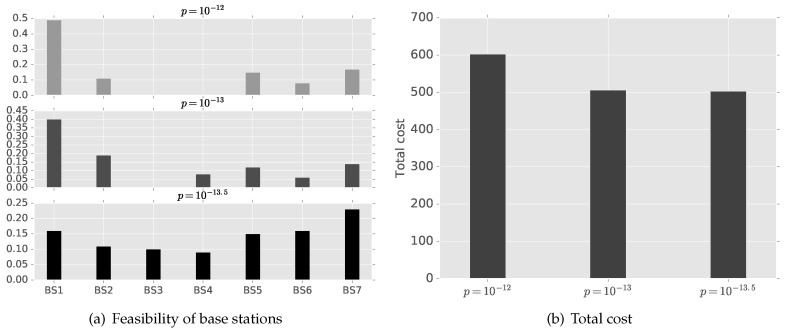
Average feasibility of base stations for cluster heads in Cell 1 with different power prices.

The cost function of the proposed model formulates energy consumption on communication and base stations’ availability (load) in Equation (6). Thus, we inspect the sensitivity of the model on base station’s availability with varying power price. The power price, *p*, represents the efficiency of the communication module. A cluster head with a more efficient communication module can lower the power price. Figure 4 shows the average feasibility of base stations for cluster heads in Cell 1 with different power prices. The cluster heads have more freedom in finding the optimal best station when the power price is low: the lower the price, the farther base stations are used, since the availability of base stations becomes a more dominant factor in the total cost.

Table 1 illustrates the equilibrium of the feasibility of base stations in Cell 1 for different power prices ($p=10^{-12}$ and $p=10^{-13.5}$). When the power price is relatively high, $p=10^{-12}$, it is beneficial to request a connection to a father base station with a smaller load than to the closest one in the TDMA system. For example, the nearest two cluster heads are connected to BS1 with 100% feasibility, but $u_{4}$ has higher feasibility for BS1 than $u_{3}$, because BS5 has more users than BS7 (lower availability). However, there is no distance-dependence on selecting base stations with a lower power price ($p=10^{-13.5}$).

**Figure 5:**
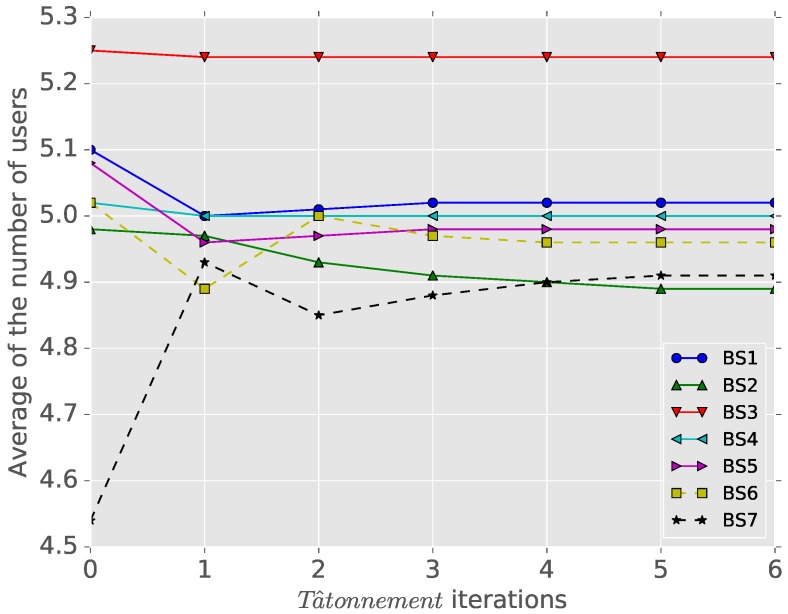
Resilience against mobile cluster heads.

We test the resilience of the proposed game model in the TDMA system with a mobile cluster. When a cluster moves from Cell 1 to an adjacent cell, we check the result of the new Tâtonnement process (from the state of Figure 3) in Figure 5. Initially, the game models of Figure 3 requires five iterations in the Tâtonnement process to converge, but we see that the disturbed game with the mobile cluster converges after two iterations. From the existence of this game solution and by defining all of the limit points (total costs) of the equilibrium for every perturbed game as a set, it is intuitive that the equilibrium of this game example is resilient against changes, since we have a finite number of users, and every perturbed game has a finite limit point [2,12].

### 4.3. CDMA Base Station

In the CDMA system, cluster head *i* has the objective function, Equation (10), and equilibrium cost, Equation (12). For the CDMA base station, we set the following parameter values: $P_{0}=\frac{10^{0.8}}{50}$, $G_{0}=64$, $N_{c}=3$, $\eta =3\times 10^{-15}$, γ=4 and $p=10^{-12}$. We assume the same power cost function and initial value for $\pi_{i,k}$ as in Section 4.2. However, unlike TDMA, cluster head *i*’s connection cost to base station *k* depends not on the static availability of base stations, but $SNIR_{i,k}$: if $SNIR_{i,k}<\gamma$, then the connection cost is infinite, and SNIR depends on the distance between the cluster heads and their base stations (see Section 3.3). The higher the distance, the lower the base stations’ SNIR and the higher the connection cost. Hence, SNIR is high and the connection cost is low when all of the users are connected to their nearest base stations.

We can get equilibrium at the initial assignment since the availability of the base station is not a static limit with the CDMA base stations (even though the base station may be dysfunctional). The power price in our initial assumption is relatively high and gives more weight to the power cost that depends heavily on the distance between cluster heads and base stations. In other words, SNIR is the most dominant factor in the initial setup. Hence, cluster heads are connected to the nearest base stations; this gives the highest SNIR for each base station, and cluster heads do not find any benefits from changing their base stations (we decrease the power price and see the game equilibrium after studying the SNIR’s of each base station).

Table 2 shows the SNIR of each cluster head in BS1 for each base station. The numbers in the table imply that SNIR’s for BS1 and BS3 are relatively low and SNIR’s for BS2 and BS7 are high. This is because of the number of cluster heads in those base stations. According to Equation (9), the SNIR’s of all of the cluster heads in the same base station are the same if they are connected to their closest base stations, and we see that the SNIR’s of all users to BS1 are the same from Table 2. According to Equation (10), there is a tradeoff between the power cost and the connection cost. For example, $u_{9}$ has a higher SNIR for BS5 than for BS1, but $u_{9}$ choose BS1 because of the power cost.

**Figure 6:**
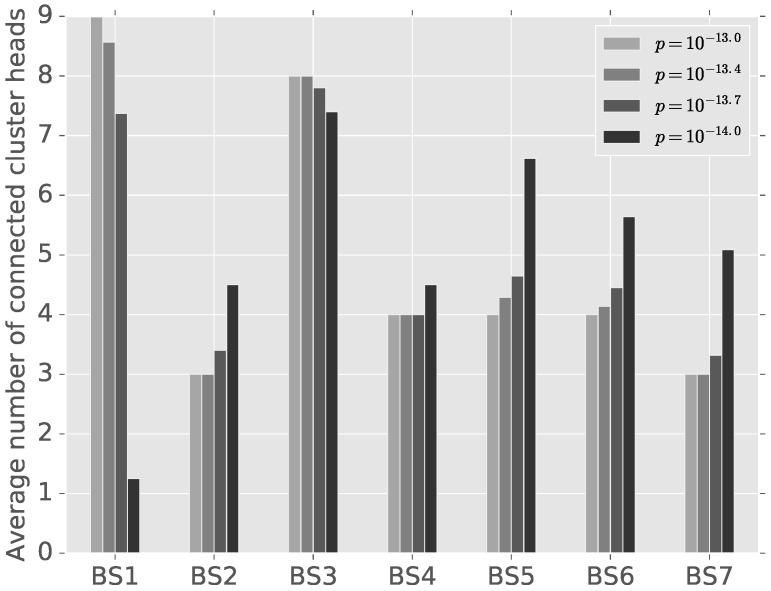
Average number of cluster heads connected to each base station with different power prices.

Figure 6 shows the optimal number of cluster heads connected to each base station at the game equilibrium in the CDMA system with different power prices. When power price decreases, cluster heads at the boundary, such as $u_{8}$ and $u_{9}$, change the optimal base station probabilities since the cost reduction from changing the base stations with high SNIR’s is greater than the additional power cost by connecting to the farther base stations. However, those boundary cluster heads’ greedy optimization decreases the SNIR’s of cluster heads not only in the new optimal base station, but also in other base stations. The lower the power price, the more freedom users have in choosing the base stations and, therefore, the lower SNIR’s. We can observe that the SNIRs of all of the base stations decrease as cluster heads choose father base stations.

**Figure 7:**
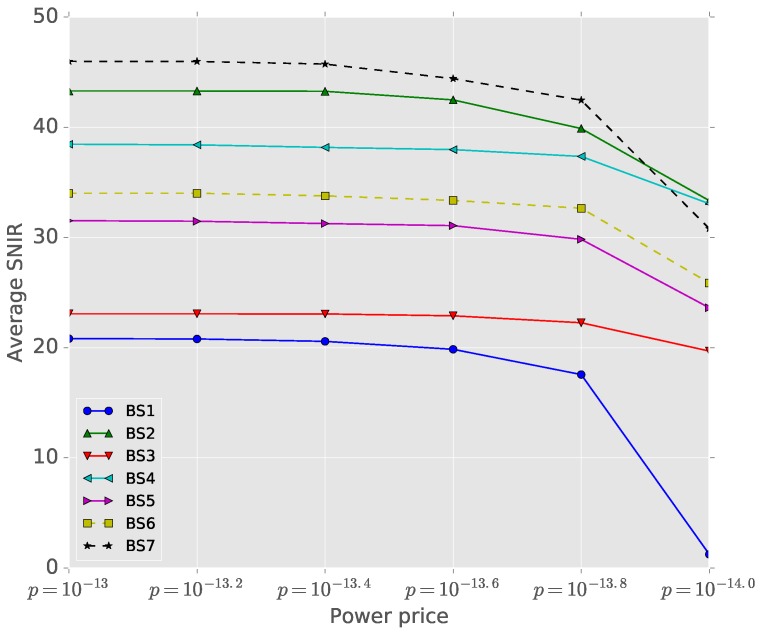
Average SNIR of all cluster heads for different power prices.

Figure 7 shows the average SNIR of all of the cluster heads under the equilibrium with different power prices. As the power price decreases, cluster heads change their base stations. When $p=10^{-14}$, the average SNIR of BS1 drops significantly because eight cluster heads in BS1 change their base stations, and the connection costs of the other cluster heads in BS1 become infinite. Finally, all of the cluster heads in BS1 choose stations other than BS1, since the SNIR is lower than *γ* (=4.0) and the capacity cost is infinite. Additionally, the whole systems’ efficiency becomes deteriorated since BS1 is dysfunctional. This shows that the power price control and coordination in CDMA systems is crucial to maintain its efficient resource allocation.

Table 3 is the feasibility vectors of cluster heads in Cell 1 when power price $p=10^{-13.5}$. Only boundary cluster heads, e.g., $u_{8}$ and $u_{9}$, have changes in the feasibility vectors. Due to SNIR, only boundary cluster heads are candidates to change the base stations, while all of the cluster heads in the TDMA system may choose different optimal base stations.

The power cost in the CDMA has a significant effect on the game equilibrium, since it decides the boundary cluster heads. In other words, the power price influences which cluster heads are candidates for boundary cluster heads. This makes the equilibrium computation and implementation of the model simple.

## 5. Related Work

Pricing and game theoretic approaches to resource allocation in communication networks have received much attention (see [15,16,17,18,19]). The research in [17,20,21] provides a pricing-based resource allocation for power control in cellular systems. There has also been efforts to formulate and study the behavior of self-interested wireless mobile users and to analyze their efforts on power control and pricing (see [19,22,23]). In this paper, we consider a related problem on the optimal base station selection in cellular systems. Amzallag et al. propose two algorithms for optimal cell selection in [24], and they formulate the optimization problem as all-or-nothing demand maximization. The main difference between our formulation and the previous models in that we allow cluster heads to pick their base stations. Each cluster head can make a decision based on the feasibility vector and its local information. Although power control issues in cellular systems have been studied extensively, the questions of the optimal base station, its benefits and implementation issues have not received the same level of attention. Our game equilibrium is derived by using a Tâtonnement process (see, e.g., [2]). The iterations in the Tâtonnement process are suitable to support dynamic changes in communication systems. According to Vives [12], the type of games that we use has a nice stability property. A related result is proven in [13]. Recently, focusing on a specific communication system, the optimization for base station deployment or cell selection has been researched. In [25,26], two algorithms are proposed to efficiently deploy LTE base stations. Likewise, Safdar et al. uses game theory to find efficient allocation for uplink communication in [27]. The heterogeneity of wireless communications makes the optimization of resource allocation difficult. In [28], Mankar et al. utilized game theory to find optimal, orthogonal spectrum allocation for a heterogeneous network composed of two-tiered cellular networks. Huang et al. focus on resource allocation in mixed cellular networks of multi-cell device-to-device communication in [29]. They handle optimizations in a specific communication system to maximize performance. However, we pay attention to providing a generalized solution for hybrid wireless sensor networks. The game theory approach is also widely employed in wireless sensor networks. To effectively cover a target field with the minimum number of sensor nodes, the game theoretic approach is used in [30,31]. Concerning the communication systems in WSN, Gao et al. present a game theoretic approach for both cell section and resource allocation in heterogeneous wireless network in [32]. However, they did not evaluate the effects of power price (the efficiency of wireless technologies). The proposed model shows that the optimization is getting crucial with more efficient wireless communication systems and can be applied to various cellular systems.

## 6. Conclusions

A hybrid WSN is a promising alternative to traditional WSN thanks to the inexpensive and widely-used cellular networks. Efficient resource allocation is unceasingly important in the divergence of cellular system. However, optimal coordination among self-interested cluster heads is complicated because base stations’ availability, SNIR and efficiency should be taken into account together. For that purpose, we assume an ideal environment to establish a logical model, and then, we employed the game theory model. We formulated the efficiency of wireless technologies as power price with the availability of the base station (for TDMA) or SNIR (for CDMA) together into a cost function. The proposed model provides optimal coordination among base stations and cluster heads. According to the result of simulation, we could reduce the total cost by 43.78% with the proposed model. Our analyses also provided interesting insights. The game theory model gives stable optimal results (equilibrium) for mobile cluster heads. In the CDMA system, base station coordination is applicable to the boundary nodes only, depending on the power price. Our method can be applied to IoT devices that communicate with limited base stations of mixed wireless technologies, as well as hybrid WSNs.

## Figures and Tables

**Table 1:** Equilibrium for cluster heads in BS1 when $p=10^{-12}$ and $p=10^{-13.5}$.

$\left(p=10^{-12}\right)$							
	**BS1**	**BS2**	**BS3**	**BS4**	**BS5**	**BS6**	**BS7**
$u_{1}$	1.00	0.00	0.00	0.00	0.00	0.00	0.00
$u_{2}$	1.00	0.00	0.00	0.00	0.00	0.00	0.00
$u_{3}$	0.74	0.00	0.00	0.00	0.00	0.00	0.26
$u_{4}$	0.81	0.00	0.00	0.00	0.19	0.00	0.00
$u_{5}$	0.59	0.00	0.00	0.00	0.41	0.00	0.00
$u_{6}$	0.00	0.00	0.00	0.00	0.00	0.00	1.00
$u_{7}$	0.02	0.98	0.00	0.00	0.00	0.00	0.00
$u_{8}$	0.00	0.00	0.00	0.00	0.00	0.72	0.28
$u_{9}$	0.19	0.00	0.00	0.02	0.79	0.00	0.00
Average	0.49	0.11	0.00	0.00	0.15	0.08	0.17
$\left(p=10^{-13.5}\right)$							
$u_{1}$	0.22	0.00	0.00	0.00	0.20	0.48	0.10
$u_{2}$	0.14	0.29	0.57	0.00	0.00	0.00	0.00
$u_{3}$	0.14	0.28	0.00	0.00	0.00	0.15	0.43
$u_{4}$	0.24	0.00	0.00	0.53	0.23	0.00	0.00
$u_{5}$	0.11	0.00	0.00	0.00	0.43	0.46	0.00
$u_{6}$	0.25	0.00	0.00	0.00	0.00	0.26	0.49
$u_{7}$	0.09	0.41	0.30	0.00	0.00	0.00	0.20
$u_{8}$	0.00	0.00	0.00	0.00	0.00	0.11	0.89
$u_{9}$	0.24	0.00	0.00	0.31	0.45	0.00	0.00
Average	0.16	0.11	0.10	0.09	0.15	0.16	0.23

**Table 2:** SNIR of cluster heads in BS1.

	BS1	BS2	BS3	BS4	BS5	BS6	BS7
$u_{1}$	22.55	41.85	22.36	37.19	30.53	32.93	44.44
$u_{2}$	22.55	42.35	22.45	37.25	30.54	32.94	44.54
$u_{3}$	22.55	42.11	22.38	37.22	30.56	33.06	45.47
$u_{4}$	22.55	41.92	22.40	37.92	32.67	33.21	44.54
$u_{5}$	22.55	41.93	22.39	37.45	32.16	33.91	44.65
$u_{6}$	22.55	43.13	22.41	37.26	30.59	33.22	50.43
$u_{7}$	22.55	46.99	23.02	37.40	30.58	32.99	44.89
$u_{8}$	22.55	42.20	22.40	37.31	30.79	37.66	49.23
$u_{9}$	22.55	41.99	22.48	42.28	35.12	33.23	44.59
Average	22.55	42.72	22.48	37.92	31.50	33.68	45.86

**Table 3:** Equilibrium for cluster heads in BS1 when $p=10^{-13.5}$.

	BS1	BS2	BS3	BS4	BS5	BS6	BS7
$u_{1}$	1.00	0.00	0.00	0.00	0.00	0.00	0.00
$u_{2}$	1.00	0.00	0.00	0.00	0.00	0.00	0.00
$u_{3}$	1.00	0.00	0.00	0.00	0.00	0.00	0.00
$u_{4}$	1.00	0.00	0.00	0.00	0.00	0.00	0.00
$u_{5}$	1.00	0.00	0.00	0.00	0.00	0.00	0.00
$u_{6}$	0.97	0.00	0.00	0.00	0.00	0.00	0.03
$u_{7}$	1.00	0.00	0.00	0.00	0.00	0.00	0.00
$u_{8}$	0.74	0.00	0.00	0.00	0.00	0.26	0.00
$u_{9}$	0.63	0.00	0.00	0.00	0.37	0.00	0.00
Average	0.93	0.00	0.00	0.00	0.04	0.03	0.00
